# Can visual cortex non-invasive brain stimulation improve normal visual function? A systematic review and meta-analysis

**DOI:** 10.3389/fnins.2023.1119200

**Published:** 2023-03-02

**Authors:** Umar M. Bello, Jingying Wang, Adela S. Y. Park, Ken W. S. Tan, Blossom W. S. Cheung, Benjamin Thompson, Allen M. Y. Cheong

**Affiliations:** ^1^Centre for Eye and Vision Research, Hong Kong Science Park, Hong Kong, Hong Kong SAR, China; ^2^Department of Physiotherapy and Paramedicine, School of Health and Life Sciences, Glasgow Caledonian University, Glasgow, United Kingdom; ^3^School of Optometry, The Hong Kong Polytechnic University, Kowloon, Hong Kong SAR, China; ^4^School of Optometry and Vision Science, University of Waterloo, Waterloo, ON, Canada

**Keywords:** non-invasive brain stimulation, visual function, meta-analyses, transcranial direct current stimulation, transcranial electrical stimulation, contrast sensitivity, visual evoked potentials, crowding

## Abstract

**Objective:**

Multiple studies have explored the use of visual cortex non-invasive brain stimulation (NIBS) to enhance visual function. These studies vary in sample size, outcome measures, and methodology. We conducted a systematic review and meta-analyses to assess the effects of NIBS on visual functions in human participants with normal vision.

**Methods:**

We followed the PRISMA guidelines, and a review protocol was registered with PROSPERO before study commencement (CRD42021255882). We searched Embase, Medline, PsychInfo, PubMed, OpenGrey and Web of Science using relevant keywords. The search covered the period from 1st January 2000 until 1st September 2021. Comprehensive meta-analysis (CMA) software was used for quantitative analysis.

**Results:**

Fifty studies were included in the systematic review. Only five studies utilized transcranial magnetic stimulation (TMS) and no TMS studies met our pre-specified criteria for meta-analysis. Nineteen transcranial electrical stimulation studies (tES, 38%) met the criteria for meta-analysis and were the focus of our review. Meta-analysis indicated acute effects (Hedges’s g = 0.232, 95% CI: 0.023–0.442, *p* = 0.029) and aftereffects (0.590, 95% CI: 0.182–0.998, *p* = 0.005) of tES on contrast sensitivity. Visual evoked potential (VEP) amplitudes were significantly enhanced immediately after tES (0.383, 95% CI: 0.110–0.665, *p* = 0.006). Both tES (0.563, 95% CI: 0.230–0.896, *p* = 0.001) and anodal-transcranial direct current stimulation (a-tDCS) alone (0.655, 95% CI: 0.273–1.038, *p* = 0.001) reduced crowding in peripheral vision. The effects of tES on visual acuity, motion perception and reaction time were not statistically significant.

**Conclusion:**

There are significant effects of visual cortex tES on contrast sensitivity, VEP amplitude, an index of cortical excitability, and crowding among normally sighted individuals. Additional studies are required to enable a comparable meta-analysis of TMS effects. Future studies with robust experimental designs are needed to extend these findings to populations with vision loss.

**Clinical trial registration:**

ClinicalTrials.gov/, identifier CRD42021255882.

## 1. Introduction

Non-invasive brain stimulation (NIBS) enables the modulation of neural activity in targeted, superficial areas of the human brain. There are two primary NIBS techniques: transcranial magnetic stimulation (TMS) and transcranial electrical stimulation (tES).

Transcranial magnetic stimulation utilizes electromagnetic induction to generate brief electric currents within the stimulated brain area and can be delivered as either single pulses or a string of repetitive pulses. Single pulses of TMS can generate action potentials that induce a motor or perceptual response. For example, TMS delivered to the primary motor cortex can cause peripheral muscle contraction (Pascualleone et al., 1994) and TMS of the primary visual cortex can induce a phosphene percept (Bohotin et al., 2003). Repetitive pulses of TMS (rTMS) can increase or decrease cortical excitability within the stimulated brain region and alter the regional concentration of neurotransmitters such as gamma-aminobutyric acid (GABA) and glutamate (Michael et al., 2003). The effect of rTMS on cortical excitability and local neurochemistry depends on the structure of the pulse train (Hallett, 2000). Commonly used pulse trains include 1 and 10 Hz stimulation frequencies as well as continuous and intermittent theta burst protocols (cTBS and iTBS) (Huang et al., 2005).

Transcranial electrical stimulation involves the delivery of an electrical current to the brain using head-mounted electrodes. tES stimulation protocols include transcranial direct current stimulation (tDCS), transcranial random noise stimulation (tRNS) and transcranial alternating current stimulation (tACS). tES does not induce action potentials but may alter membrane potentials [tDCS (Nitsche and Paulus, 2001; Nitsche et al., 2003), tRNS (Terney et al., 2008)], induce regional changes in neurotransmitter concentration [tDCS (Stagg et al., 2009; Bachtiar et al., 2015; Hunter et al., 2015)], alter cortical excitability [tDCS (Nitsche and Paulus, 2000, 2001), tRNS (Terney et al., 2008; Moliadze et al., 2010)], entrain patterns of neural activity [tACS (Battleday et al., 2014)] and alter the signal to noise ratio within stimulated regions [tRNS, refer to Reed (Reed and Kadosh, 2018) for a review]. NIBS has been used in multiple research contexts including the study of fundamental neurological processes, cognition (Hoy et al., 2015; Grabner et al., 2018; Guleken et al., 2020), and the development of new therapeutic interventions [e.g., depression (Martin et al., 2018; Moreno et al., 2020), neurorehabilitation (Liew et al., 2014)].

Visual brain areas are attractive targets for NIBS research because regions such as the primary visual cortex and motion sensitive extrastriate area [middle temporal (MT)] are close to the cortical surface and techniques such as visual psychophysics, electroencephalography, and magnetic resonance imaging are available to measure the effects of the stimulation on neural activity and perception (Thompson et al., 2009, 2016; Miniussi et al., 2013). In addition, NIBS is emerging as a promising tool for vision rehabilitation (Pascual-Leone et al., 1998; Thompson et al., 2008). However, the literature on NIBS of visual brain areas is diverse with a wide range of different study designs, stimulation protocols, outcome measures and population samples. The aim of this structured review and meta-analysis was to assess whether visual cortex NIBS can enhance visual perception and/or modulate visual cortex activity (measured using visual evoked potentials). We did not include studies that used NIBS to induce “virtual lesions” or impair visual function to probe fundamental neurological processes. Our original plan was to review visual cortex NIBS studies involving either healthy or clinical populations [e.g., amblyopia (Spiegel et al., 2013; Ding et al., 2016) or hemianopia (Plow et al., 2012; Olma et al., 2013)]. However, our literature search revealed that studies of clinical populations did not employ common study designs and were relatively few. We therefore limited our review to studies examining the effect of NIBS on vision enhancement in healthy participants with normal vision.

## 2. Materials and methods

This systematic review conforms to the Preferred Reporting Items for Systematic Reviews and Meta-Analyses 2020 (PRISMA-2020) guidelines (Page et al., 2021). We registered the review protocol with the International Prospective Register of Systematic Reviews (PROSPERO; Ref. No: CRD42021255882) in June 2021, prior to the initiation of the data extraction processes. We adopted the PICO (Participants, Intervention, Comparators and Outcome) format in generating the research question. The intervention was any form of NIBS (including tDCS, tACS, tRNS, and TMS), while the comparators included sham (placebo) NIBS. Outcomes of interest included psychophysical measures of contrast sensitivity, visual crowding, visual acuity, motion perception, visual evoked potentials (VEPs), and reaction time among others. The study conceptualization and development of the review protocol were undertaken by authors UMB, JYW, BT, and AMYC.

### 2.1. Search strategy

A systematic search of PubMed, Embase, PsycINFO, Web of Science, Medline and OpenGrey databases was conducted from 1st January 2000 until 1st September 2021. The search terms were grouped under two themes, namely: “Brain area,” and “NIBS.” The electronic search involved combining terms under each theme using the Boolean operator “OR.” The search themes were then combined using the Boolean “AND” (see Supplementary material for details of the search themes/terms). Citation management software (EndNote X9, Clarivate Analytics, Philadelphia, PA, USA) was used to organize the electronic search results and remove duplicates. Two of the authors (JYW and UMB) independently conducted the electronic search. Any discrepancies during the independent search process were resolved by consulting a third author (AMYC). A thorough manual search of the reference lists of the identified studies and a forward reference search (*via* Google scholar) were also conducted.

### 2.2. Study eligibility criteria

Studies were included if they: (i) assessed the effect of NIBS on enhancing visual functions among normally sighted individuals; (ii) included a sham stimulation control; (iii) were available in full text and (iv) written in English. We excluded studies that were: (i) conducted on individuals presenting with mental disorders, cognitive impairments, or visual impairments; (ii) used NIBS to disrupt or impair visual function, (iii) review protocols; (iv) systematic reviews; (v) conference abstracts and (vi) case studies.

### 2.3. Article screening

The identified studies *via* electronic search processes were sequentially screened at the title, abstract and full text phases by two of the authors (JYW and UMB). Any discrepancies identified by the two authors during the screening phases were resolved by discussion or consultation with the corresponding author (AMYC).

### 2.4. Data extraction

The primary data for this study quantified the effect of NIBS on enhancing visual functions. Other relevant data extracted included the study reference, year of publication, title of study, study design, NIBS method, brain area stimulated, and visual function(s) measured. Data extraction was undertaken independently by JYW and BWSC using an extraction tool designed in Microsoft Excel. Disagreements between the authors during the data extraction process were resolved by discussion or consultation with the other authors (BT and AMYC).

### 2.5. Data analysis

Meta-analyses were conducted using the Comprehensive Meta-Analysis (CMA) software version 3.0 (Biostat Inc., Englewood, NJ, USA). Outcomes of studies that utilized protocols from the same NIBS delivery technique (tES or TMS) and reported findings on the same visual function were pooled for meta-analyses. Therefore tDCS, tRNS, and tACS studies were pooled and rTMS and TBS studies were pooled. Similar studies with differing techniques for measuring a specific visual function could be pooled. Finally, studies with a common outcome measure were pooled. Examples include reaction time and VEP. Within each pooled group, we included all relevant studies and looked at *acute* (immediate, same day pre- vs. post-effects of NIBS), and *after* effects (same day, but at a designated time point after stimulation—i.e., 10–30 min post-stimulation). In the first instance, all related NIBS subtypes (tES or TMS) were combined for a general overview, and where there were enough studies, the stimulation protocol subtypes were analyzed separately. Studies numbering two and above that met the meta-analysis criteria were pooled in a meta-analysis, in-line with a previous recommendation (Valentine et al., 2010). Study authors were contacted via email to obtain any missing data for the included studies. Unless otherwise indicated, stimulation was applied to the occipital lobe/primary visual cortex (V1). Data presented graphically were extracted using the GetData Graph Digitizer 2.26.^1^ Data reported as median and range were converted to mean and standard deviation (Hozo et al., 2005). We adopted the bias-adjusted, standardized mean difference (SMD; Hedges’s g) to analyze the extracted data from the primary studies. The chi-square test (I^2^ statistics) was used to determine the degree of variance across studies (Higgins et al., 2003), and a random-effects model was used for all the meta-analyses due to methodological heterogeneity among the studies. A *p*-value of <0.05 indicated statistical significance.

### 2.6. Quality appraisals of the included studies

Two authors (ASYP, KWST) attempted to conduct quality ratings of the included studies using the Downs and Black quality rating tool (Downs and Black, 1998), which consisted of 27 items. Ratings were conducted independently prior to comparison. However, it was noted that 14 randomly selected studies, were all rated of “poor” quality, suggesting that perhaps this instrument might not have been the most appropriate for the types of intervention studies included in this meta-analysis.

## 3. Results

### 3.1. Characteristics of the included studies

In total, 5,266 studies were identified through the electronic database and manual searches, among which 50 met the review criteria after sequential screening of the title, abstract and the full text (Kraft et al., 2010; Fertonani et al., 2011; Zito et al., 2015; Barbieri et al., 2016; Ding et al., 2016; Reinhart et al., 2016; van Koningsbruggen et al., 2016; Battaglini et al., 2017, 2020a; Behrens et al., 2017; Bonder et al., 2018; Contemori et al., 2019; He et al., 2019; Dong et al., 2020; Nakazono et al., 2020; Raveendran et al., 2020; Wu et al., 2020; Chen et al., 2021; Lau et al., 2021). Of these studies, 19 met the criteria for meta-analysis. Supplementary material summarizes the reasons why the remaining 31 studies were not eligible for meta-analysis. Only five TMS studies were identified through the search process. Because these studies did not have common methodologies or outcomes measures, they did not meet our criteria for meta-analysis. Therefore, our meta-analysis was conducted only on tES studies.

**FIGURE 1:**
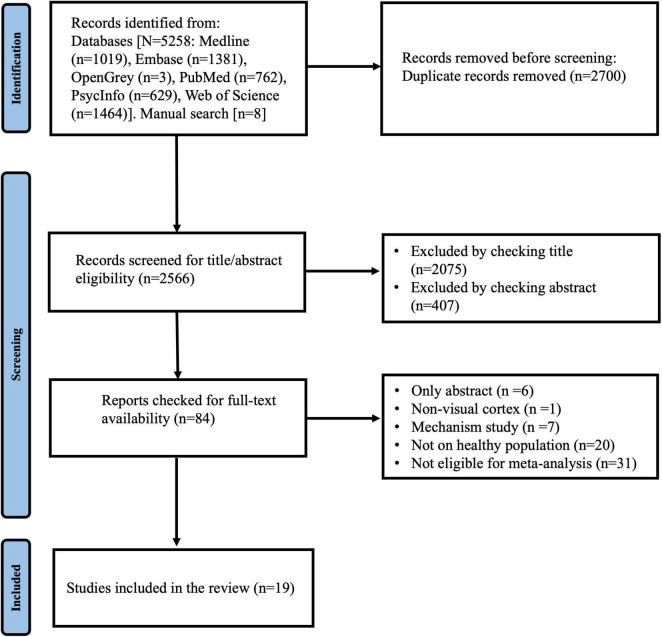
Study flowchart.

The study flowchart detailing the search outcome and screening processes is presented in Figure 1. Overall, the included studies recruited 674 participants. For the NIBS modalities adopted in the included studies, most studies utilized tDCS (*n* = 14, 73.7%), then tRNS (*n* = 3, 15.8%) and tACS (*n* = 1, 5.3%). Another study utilized tRNS with tDCS (*n* = 1, 5.3%). The visual functions examined among the studies were contrast sensitivity (*n* = 7, 36.8%), reaction time (*n* = 6, 31.6%), VEPs (*n* = 4, 21.1%), motion perception (*n* = 3, 15.8%), crowding (*n* = 3, 15.8%), and visual acuity (*n* = 2, 10.5%)^2^. Table 1 presents the study characteristics.

**TABLE 1:** Characteristics of the included studies (*n* = 19).

S/ No	Study references	Study design	Outcome measures	Age (years)	Sex (m:f)	*N*	NIBS	Stimulation site	Online/ Offline	Montage (target-ref/target)	Neuro	Duration (min)	Stimulation sessions (n)	Intensity (mA/ MSO)	Size of electrode/Coil (cm2; target-reference)	Density (mA/ cm2)	Stimuli	Side-effect
1	Barbieri et al., 2016	Between subjects, sham controlled	Face perception, object perception (RT)	27	15:33	48	atDCS	Occipito-temporal cortex	Online+ offline	PO8-FP1	Yes, EEG	Online: 24.6 Sham: 24.6 Offline: 20	1	1.5	25–25	0.08	Faces, objects	No side effect
2	Battaglini et al., 2017	Within-subjects, sham controlled	Motion perception	/	15:15	30	atDCS, ctDCS	V5	Offline	Left V5/MT-Cz	No	12	4	1.5	5*7–5*7	0.043	Moving dots	/
3	Battaglini et al., 2020a	Within-subjects, sham controlled	CS	25 ± 3.4	7:13	20	tRNS	V1	Online	Oz–Cz	/	15	4	1.5	7.2*6–11.5*9.5	0.03–0.01	Gabor patches	Mild skin sensation
4	Behrens et al., 2017	Between-subjects, sham controlled	CS	24.5 ± 3.5	12:12	24	atDCS	V1	Offline	V1–Cz	yes, MRI	20	5	1.5	/- 7*5	0.06	Humphrey perimetry (central 10^°^)	/
5	Bonder et al., 2018	Between-subjects, sham controlled	VA (RT)	26.3	8:22	30	atDCS	Occipital cortex	Online	O1-FP2	Yes, EEG	15	1	1	5*5–7*5	0.04–0.03	A black square with a gap on either side	
6	Chen et al., 2021	Between subjects, sham controlled	Crowding	/	Exp 1: 23:22; Exp 2: 21:24; Exp 3: 16:12	118	atDCS	Occipital cortex	Offline	P1/P2–ipsilateral cheek	Yes, EEG	20	1	2	35–35	0.06	Gratings, sloan letters	/
7	Contemori et al., 2019	Between-subjects, sham controlled	Crowding	25	15:17	32	tRNS (100–640 Hz)	Occipital cortex	Online	Oz–Cz	No	30	4	1.5	16–27	0.094	Single white letter, crowded white letters	/
8	Ding et al., 2016	Within-subjects, sham controlled	CS, VEPs	23 ± 2.3	/	27	atDCS, ctDCS	Occipital cortex	Online + offline	Oz–Cz	Yes, EEG	20	6	2	4*6–5*7	0.083–0.057	Gabor patches, pattern-reversal checkerboards	/
9	Dong et al., 2020	Within-subjects, sham controlled	EEG	18–26	10:5	15	atDCS	Occipital cortex	Offline	Oz–Cz	Yes, EEG	21	2	2	35–35	0.06	White cross fixation	No side effect
10	Fertonani et al., 2011	Between-subjects, sham controlled and between-subjects, non-sham controlled	Orientation discrimination (RT)	21.7 ± 2.5	42:42	84	lf-tRNS (0.1–100 Hz), hf-tRNS (100–640 Hz), atDCS; ctDCS	V1	Online	Oz–right arm	Yes, EEG	22	1	1.5	16–60	0.025–0.06	Tilted black lines	tDCS-induced sensations were perceived stronger
11	He et al., 2019	Within-subjects, sham controlled	CS	23.4 ± 1.9	16:11	27	atDCS	Occipital cortex	Offline	Oz–Cz	Yes, EEG	15	3	2	5*5–5*5	0.08	Gratings	No side effect
12	Kraft et al., 2010	Within subjects, sham controlled	CS	25.9 ± 1.83	5:7	12	atDCS, ctDCS	Occipital cortex	Offline	O1/O2–Cz	Yes, MRI	15	3	1	5*5–7*10	0.04–0.014	Humphrey perimetry	/
13	Lau et al., 2021	Within-subjects, sham controlled	VEPs	28.7	6:14	20	atDCS, ctDCS	V1	Offline	Oz–Cz	Yes, EEG	20	3	2	5*7–5*7	0.06	Pattern-reversal checkerboard	/
14	Nakazono et al., 2020	Exp 1: single arm Exp 2 and 3: within-subjects, sham-controlled	CS, EEG, VEPs	26.8 ± 7.8	Exp 1: 6:7 Exp 2: 10:7 Exp 3: 7:8	Exp 1: 13 Exp 2: 17 03: 00 Exp 3: 15	tACS (10, 20 Hz)	V1	Offline	Oz–Cz	Yes, EEG	20	Exp 1: 2 Exp 2 and 3: 3	1	3.5*3.5–7*5	0.08–0.03	Pattern-reversal checkerboard, reversing black and white fields, Gabor patches	Itching, flickering
15	Raveendran et al., 2020	Within-subjects, sham controlled	Crowding	/	/	13	atDCS	V1	Online	Oz–Cz	Yes, EEG	20	2	2	5*5–5*5	0.08	Gabor patches with flankers	/
16	Reinhart et al., 2016	Exp 1: single arm Exp 2–5: within subjects, sham controlled	Motion perception, VA, VEPs (RT)	Exp 1: 22.0 ± 0.9 Exp 2: 25.3 ± 1.3 Exp 3: 23.1 ± 1.2; Exp 4: 22.4 ± 1.3; Exp 5: 20.4 ± 1.8	Exp 1: 9:11 Exp 2: 13:7 Exp 3: 7:13 Exp 4: 10:10 Exp 5: 14:6	20	atDCS, ctDCS	Occipito- parietal cortex	Offline	Exp 1: P1/P2-left/right cheek (ipsilateral) Exp 2: left/right cheek (ipsilateral)-P1/P2 Exp 3: C3/C4-left/right cheek (ipsilateral) Exp 4–5: P1/P2-left/right cheek (ipsilateral)	Yes, EEG	20	Exp 1: 3 Exp 2–5: 2	Exp 1: 1/1.5/2 Exp 2–5: sham/2	19.25–52	0.1–0.038	Vernier stimulus, snellen letters, gratings	Tingling, itching
17	van Koningsbruggen et al., 2016	Between subjects, sham controlled	Attentional capture (RT)	23.8 ± 3.6	20:40	60	tRNS (100–640 Hz)	Lateral occipital cortex	Online	PO7–PO8	Yes, EEG	20	1	1	5*7–5*7	0.03	Black lines in empty coloured circles	/
18	Wu et al., 2020	Between subjects, sham controlled	Motion perception	/	28:0	28	atDCS	V5	Offline	V5-Cz	No	20	1	1.5	5*7–5*7	0.043	Moving white dots	/
19	Zito et al., 2015	within-subjects, sham controlled	motion perception (RT), shape perception	30.5 ± 5.1	11:10	21	aHD-tDCS, cHD-tDCS	V5	Offline	P4, OZ, TP8, and PO10-PO8	Yes, EEG	20	3	2	/	/	Moving dots, ellipses	No effect

Ding et al. (2016) included participants with amblyopia and healthy controlled (but only healthy controlled were considered in this meta-analysis); N, sample size.

Outcome measures: CS, contrast sensitivity; RT, reaction time; VA, visual acuity; VEPs, visual evoked potentials.

Types of stimulation:

(1) tES, transcranial electrical stimulation; atDCS, anode transcranial direct current stimulation; ctDCS, cathode transcranial direct current stimulation; aHD-tDCS, anodal high-definition transcranial direct current stimulation; cHD-tDCS, cathode high-definition transcranial direct stimulation; tACS, transcranial alternating current stimulation; tRNS, transcranial random noise stimulation; lf-tRNS, low-frequency transcranial random noise stimulation; hf-tRNS, high-frequency transcranial random noise stimulation. (2) TMS, transcranial magnetic stimulation; rTMS repetitive transcranial magnetic stimulation; cTBS, continuous theta burst stimulation.

Application of neuro-navigation (neuro): EEG, electroencephalogram; MRI, magnetic resonance imaging.

Site of stimulation: Cz, central zero; FP, frontal pole; MT, middle temporal visual area; Oz, occipital zero; PO, posterior occipital; TP, temporal pole; V1, primary visual cortex.

### 3.2. Quantitative analysis of whether NIBS can enhance visual function

#### 3.2.1. Contrast sensitivity

##### 3.2.1.1. Acute effect of tES (a-tDCS, tRNS, and tACS) on contrast sensitivity

**FIGURE 2:**
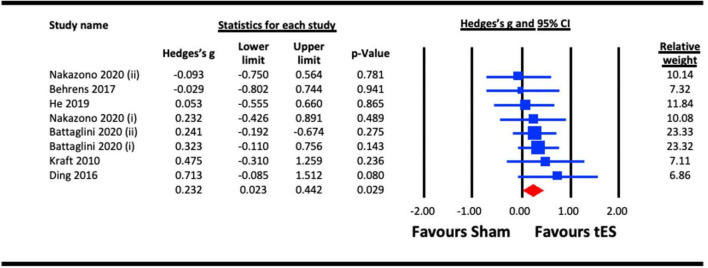
Acute effect of transcranial electrical stimulation (tES) [anodal-transcranial direct current stimulation (a-tDCS), transcranial random noise stimulation (tRNS), and transcranial alternating current stimulation (tACS)] on contrast sensitivity. Meta-analyses for Nakazono (2020) were separated for data on 9.0 cpd alpha (acute) and 9.0 cpd beta (acute), represented as Nakazono (2020) (i) and Nakazono (2020) (ii) Meta analyses for Battaglini (2020a) were separated for data using contrast stimuli of 45^°^ and vertical, represented as Battaglini (2020a) (i) and Battaglini (2020a) (ii).

Included studies measured the same-day effects of a single NIBS session on contrast sensitvity. The pooled analysis involved six studies, among which four utilized a-tDCS (Kraft et al., 2010; Ding et al., 2016; Behrens et al., 2017; He et al., 2019), one adopted tACS (Nakazono et al., 2020) and one utilized tRNS (Battaglini et al., 2020a). In studies that measured multiple outcomes, only the contrast sensitivity results were included (Ding et al., 2016; Nakazono et al., 2020). Contrast sensitivity was measured using perimetry (Kraft et al., 2010; Behrens et al., 2017), a 10 cycles per degree (cpd) Gabor patch (Ding et al., 2016), or stimuli presented at a range of spatial frequencies (He et al., 2019; Battaglini et al., 2020a; Nakazono et al., 2020). If the study measured contrast sensitivity at more than one spatial frequency, the results for the highest spatial frequency was chosen, because the most challenging condition was expected to show the greatest NIBS-induced enhancement. Spatial frequencies selected included 9 cpd (Nakazono et al., 2020), 10 cpd (Ding et al., 2016), and both 7 cpd and 12 cpd for the study by Battaglini et al. (2020a) because the authors explicitly hypothesized that sensitivity for both higher spatial frequencies would be enhanced by the tRNS. For studies that measured contrast sensitivity at more than one retinal eccentricity, the measures for central vision were selected for meta-analysis to provide consistency across studies. Nakazono et al. (2020) compared alpha and beta tACS to a sham condition. Both stimulation frequencies were included in the meta-analysis. Similarly, Battaglini et al. (2020a) used vertical and 45^°^ oriented Gabors for their contrast detection tasks. Both orientations were included in the meta-analysis. We pooled the effect of active stimulation against sham conditions for the analysis (Figure 2). The result indicated a statistically significant acute effect of tES stimulation (Hedges’s g = 0.232, 95% CI: 0.023–0.442, *p* = 0.029) on contrast sensitivity in normally sighted participants.

##### 3.2.1.2. Acute effect of anodal tDCS (a-tDCS) on contrast sensitivity

**FIGURE 3:**
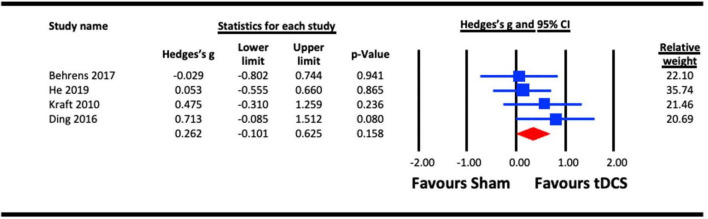
Acute effect of anodal-transcranial direct current stimulation (a-tDCS) on contrast sensitivity.

A single session acute effect of a-tDCS on contrast sensitivity is illustrated in Figure 3. Of those studies included in section 1.1, the a-tDCs studies were pooled for the analysis (Kraft et al., 2010; Ding et al., 2016; Behrens et al., 2017; He et al., 2019). There was a trend favoring an effect of a-tDCS stimulation on contrast sensitivity as per the main analysis, but this failed to reach statistical significance (Hedges’s g = 0.262, 95% CI: −0.101 to 0.625, *p* = 0.158).

##### 3.2.1.3. Aftereffect of tES (a-tDCS and tACS) on contrast sensitivity

**FIGURE 4:**
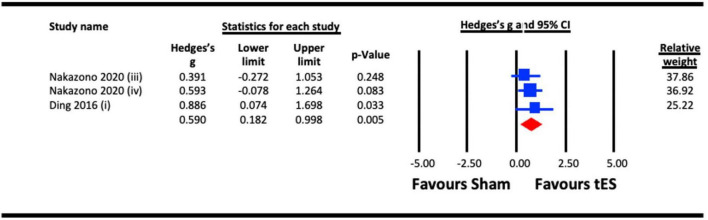
Aftereffect of transcranial electrical stimulation (tES) [anodal-transcranial direct current stimulation (a-tDCS) and transcranial alternating current stimulation (tACS)] on contrast sensitivity. Meta-analyses for Nakazono (2020) were separated for data on 9.0 cpd beta (at 10 min post) and 9.0 cpd alpha (at 10 min post), represented as Nakazono (2020) (iii) and Nakazono (2020) (iv).

Nakazono et al. (2020) and Ding et al. (2016) reported aftereffects of tES on contrast sensitivity measured 10- and 30 min post-stimulation, respectively (Figure 4). A meta-analysis revealed a statistically significant aftereffect of tES stimulation on contrast sensitivity (Hedges’s g = 0.590, 95% CI: 0.182–0.998, *p* = 0.005).

#### 3.2.2. Visual evoked potentials (VEPs)

##### 3.2.2.1. Acute effect of tES (a-tDCS and tACS) on VEP amplitude

**FIGURE 5:**
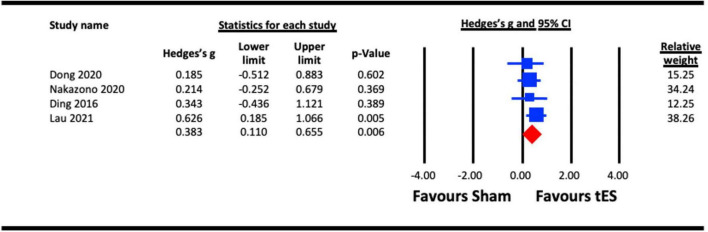
Acute effect of transcranial electrical stimulation (tES) [anodal-transcranial direct current stimulation (a-tDCS) and transcranial alternating current stimulation (tACS)] on visual evoked potentials (VEPs). Nakazono (2020), combined effect of alpha and beta tACS; Lau (2021), combined effect of N1 and P1 amplitudes.

Four studies were pooled for analysis to assess the acute effect of tES on VEP amplitude, three utilized a-tDCS (Ding et al., 2016; Dong et al., 2020; Lau et al., 2021) and one adopted tACS (Nakazono et al., 2020). In studies that measured the acute effects of NIBS on different visual functions (e.g., contrast sensitivity and VEPs), the results from the VEP measure were taken (Ding et al., 2016; Nakazono et al., 2020). Different components of VEPs were estimated in the studies, including amplitude of P100-N75 (Ding et al., 2016; Nakazono et al., 2020), amplitude of the alpha activity over the parieto-occipital area (Dong et al., 2020), and N1 and P1 amplitudes (Lau et al., 2021) (both included in the analysis). Similarly, where alpha and beta tACS were utilized in a study (Nakazono et al., 2020), the effect of each stimulation condition against a sham effect was extracted for the analysis. We pooled the effect of active stimulation against sham conditions for the analysis (Figure 5). The result indicated a statistically significant increase in VEP amplitude immediately after tES at visual cortex (Hedges’s g = 0.383, 95% CI: 0.110–0.655, *p* = 0.006).

#### 3.2.3. Crowding

##### 3.2.3.1. Acute effect of tES (a-tDCS and tRNS) on crowding

**FIGURE 6:**
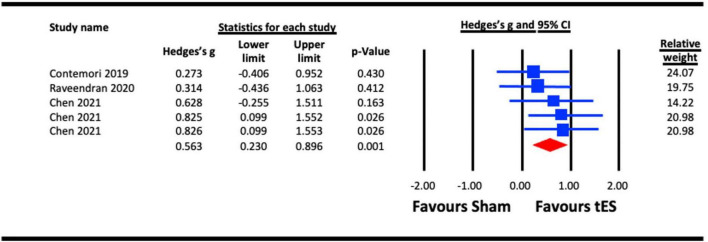
Acute effect of transcranial electrical stimulation (tES) [anodal-transcranial direct current stimulation (a-tDCS) and transcranial random noise stimulation (tRNS)] on crowding. Chen (2021) (each line represents the outcome of experiments 1–3).

Three studies were pooled for analysis to assess the acute effect of tES on crowding, two utilized a-tDCS (Raveendran et al., 2020; Chen et al., 2021), and one adopted tRNS (Contemori et al., 2019). In the study with multiple experiments involving different groups of participants (Chen et al., 2021), data from each experiment were pooled separately in the analysis. The results for NIBS applied to the hemisphere contralateral to the presented stimuli against a sham condition were pooled in the analysis (Chen et al., 2021). The earliest effect of a-tDCS on crowding (5 min post-stimulation) reported by Raveendran et al. (2020) was pooled for analysis. The analysis (Figure 6) indicated a statistically significant effect of tES on crowding (Hedges’s g = 0.563, 95% CI: 0.230–0.896, *p* = 0.001).

##### 3.2.3.2. Acute effect of a-tDCS on crowding

**FIGURE 7:**
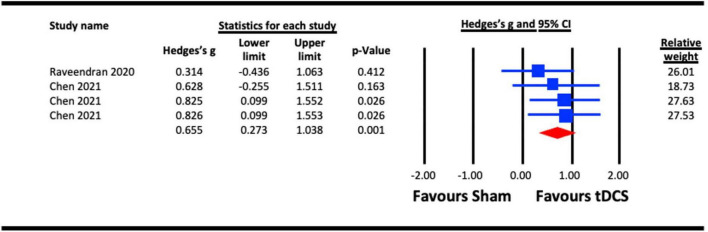
Acute effect of anodal-transcranial direct current stimulation (a-tDCS) on crowding. Chen (2021) (each line represents the outcome of experiments 1–3).

To assess the acute effect of a-tDCS on crowding (independent of tRNS), data from the two studies that used a-tDCS (Raveendran et al., 2020; Chen et al., 2021) were pooled for analysis. The result (Figure 7) indicated a statistically significant effect of a-tDCS on crowding (Hedges’s g = 0.655, 95% CI: 0.273–1.038, *p* = 0.001).

#### 3.2.4. Visual acuity

##### 3.2.4.1. Acute effect of a-tDCS on visual acuity

**FIGURE 8:**
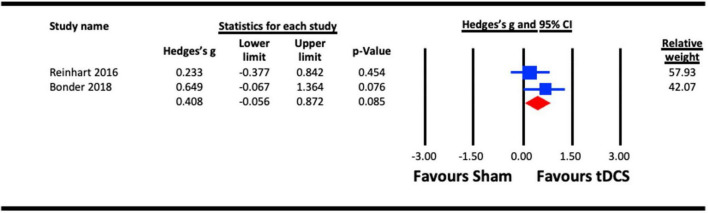
Acute effect of anodal-transcranial direct current stimulation (a-tDCS) on visual acuity.

To assess the acute effect of a-tDCS on visual acuity, two studies (Reinhart et al., 2016; Bonder et al., 2018) were pooled for analysis. In a study that measured the acute effects of a-tDCS on different visual functions (visual acuity, contrast sensitivity and VEPs), the results from the visual acuity measure were taken (Reinhart et al., 2016). The pooled effect for the active stimulation condition in each study was compared to sham conditions (Figure 8). The result indicated a statistically non-significant effect of a-tDCS on visual acuity (Hedges’s g = 0.408, 95% CI: −0.056 to 0.872, *p* = 0.085).

#### 3.2.5. Motion perception

##### 3.2.5.1. Acute effect of a-tDCS on motion perception

**FIGURE 9:**
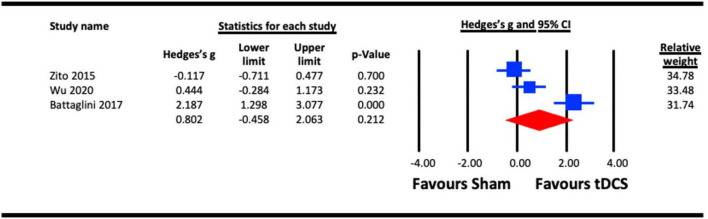
Acute effect of anodal-transcranial direct current stimulation (a-tDCS) on motion perception.

The pooled analysis to assess the acute effect of a-tDCS on motion perception involved three studies (Zito et al., 2015; Battaglini et al., 2017; Wu et al., 2020). All studies stimulated extrastriate cortical area V5 (MT). In the study that measured the acute effects of a-tDCS on different visual functions (motion and shape perception), the results from the motion perception measure were taken (Zito et al., 2015). The pooled effect for each of the study were compared against sham control conditions (Figure 9). The result indicated a statistically non-significant effect of a-tDCS on motion perception (Hedges’s g = 0.802, 95% CI: −0.458 to 2.063, *p* = 0.212).

#### 3.2.6. Reaction time

##### 3.2.6.1. Acute effect of tES (tRNS and a-tDCS) on reaction time

**FIGURE 10:**
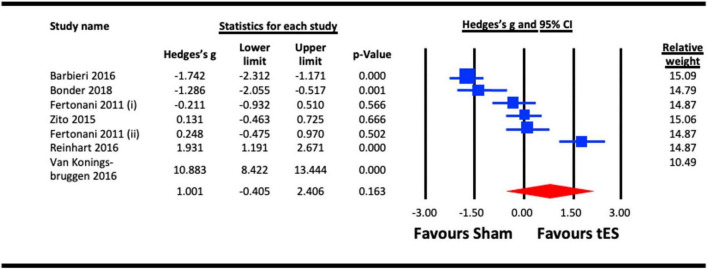
Acute effect of transcranial electrical stimulation (tES) [anodal-transcranial direct current stimulation (a-tDCS) and transcranial random noise stimulation (tRNS)] on reaction time. Barbieri (2016) (included data for combined effect of a-tDCS face and object tasks); Meta-analysis for Fertonani (2011) were separated for data on a-tDCS and tRNS, represented as Fertonani (2011) (i) and Fertonani (2011) (ii).

Reaction time was analysed as a proxy of vision-related cognitive processing. Six studies were pooled for the analysis to assess the acute effect of tES on reaction time, five utilized a-tDCS (Zito et al., 2015; Barbieri et al., 2016; Reinhart et al., 2016; Bonder et al., 2018) and two adopted tRNS (Fertonani et al., 2011; van Koningsbruggen et al., 2016). In studies that measured the acute effects of NIBS on different visual functions (e.g., face/object/motion perception, visual acuity, VEPs, contrast sensitivity, attentional capture and reaction time), the results from the reaction time measure were taken (Fertonani et al., 2011; Zito et al., 2015; Barbieri et al., 2016; Reinhart et al., 2016; van Koningsbruggen et al., 2016; Bonder et al., 2018). When a study reported the effect of multiple NIBS protocols on same group of participants (for example tRNS and a-tDCS) (Fertonani et al., 2011), both effects were pooled in the tES meta-analysis (Figure 10), respectively. Similarly, in a study with dual experimental/stimulation conditions (face and object recognition) (Barbieri et al., 2016), the effects of both conditions on the reaction time were combined and pooled in the analysis. Outcome of the analysis illustrated a statistically non-significant effect of tES on reaction time (vision-related cognitive processing) (Hedges’s g = 1.001, 95% CI: −0.405 to 2.406, *p* = 0.163).

##### 3.2.6.2. Acute effect of a-tDCS on reaction time

**FIGURE 11:**
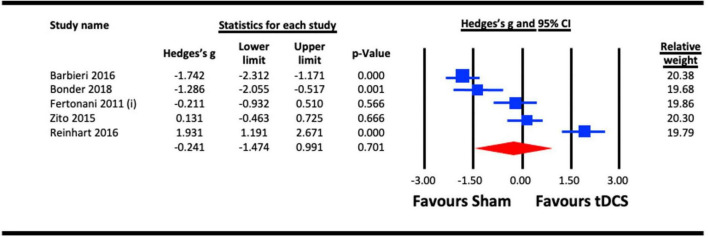
Acute effect of anodal-transcranial direct current stimulation (a-tDCS) on reaction time. Barbieri (2016), combined effect of a-tDCS face and object tasks; Fertonani (2011) (i), effect of a-tDCS.

To assess the acute effect of a-tDCS on reaction time, data from the five studies that used a-tDCS in the sub-section 6.1 (Fertonani et al., 2011; Zito et al., 2015; Barbieri et al., 2016; Reinhart et al., 2016; Bonder et al., 2018) were pooled. The result (Figure 11) indicated a statistically non-significant effect of a-tDCS on reaction time (Hedges’s g = −0.241, 95% CI: −1.474 to 0.991, *p* = 0.701).

## 4. Discussion

To recapitulate, the aim of this structured review and meta-analysis was to assess whether visual cortex NIBS could enhance visual function and/or modulate visual cortex activity. Both tES and TMS have been used as rehabilitation tools to enhance a variety of neural functions including cognition (Hara et al., 2021) and motor control (Liew et al., 2014). Unexpectedly, our literature review identified only five studies that investigated the use of TMS to enhance a specific visual function. Because these five studies used different stimulation protocols and/or different outcome measures, they could not be meta-analyzed (see Supplementary material for details). Although TMS has been widely used in vision research, it appears to have been used primarily to explore the function of targeted cortical areas or neural networks rather than a tool to enhance specific visual functions. However, it is clear that TMS does exert an effect on brain areas involved in visual processing and the five studies identified by our review reported improvements or alterations in the targeted visual function post stimulation. These effects were associated with changes in cortical excitability, neurotransmitter concentrations and signal to noise within the stimulated area. Furthermore, studies involving clinical populations have reported improvements in a variety of visual functions following visual cortex TMS (Thompson et al., 2008; Clavagnier et al., 2013). Therefore, it is likely that TMS will play a larger role in vison enhancement studies as the field continues to grow.

For the reasons described above, our meta-analysis involved only tES studies. Studies exploring the use of tES to enhance normal vision have employed a diverse range of experimental designs with time scales that range from the acute effects of a single tES session to multi-session studies that combine tES with perceptual learning. This diversity resulted in limited opportunities for meta-analyses. However, by pooling across different tES stimulation protocols and differing methodologies for assessing a common outcome measure, we were able to assess the effects of a single tES session vs. sham stimulation on contrast sensitivity, VEP amplitude, visual crowding, visual acuity, motion perception, and reaction time.

Both contrast sensitivity (Kraft et al., 2010; Ding et al., 2016; Behrens et al., 2017; He et al., 2019; Battaglini et al., 2020a; Nakazono et al., 2020) and visual crowding (Contemori et al., 2019; Raveendran et al., 2020; Chen et al., 2021) were significantly enhanced by visual cortex tES relative to sham within our meta-analyses, and were examined at different timescales relative to stimulation. Results from our meta-analyses showed beneficial acute effects of tES in enhancing contrast sensitivity (Figure 2) and reduced crowding (Figure 6). The sub-analysis of studies that only employed a-tDCS revealed improvements in crowding following stimulation (Figure 7), but not for contrast sensitivity (Figure 3). Our meta-analyses looking at later time points (i.e., aftereffects) could only be performed for contrast sensitivity as there was only one study investigating the effect of NIBS on crowding. We observed that tES was effective in modulating contrast sensitivity at a fixed time point after stimulation (Figure 4), indicating that the effects of stimulation on improving contrast sensitivity persisted beyond the stimulation period. Despite only one study measuring the aftereffects of a-tDCS on crowding (Raveendran et al., 2020) (in this case lateral-inhibition, a low-level mechanism that may contribute to crowding), the study reported a larger effect at 30 min vs. 5 min after stimulation, which is in line with the time-scale of the effects observed in the contrast sensitivity meta-analysis.

From a mechanistic perspective, the meta-analysis of changes in VEP amplitude (Ding et al., 2016; Dong et al., 2020; Nakazono et al., 2020; Lau et al., 2021) following visual cortex tES vs. sham revealed enhanced cortical excitability (i.e., larger VEP amplitudes) following tES (Figure 5). Increased cortical excitability may enhance neural sensitivity to contrast and weaken lateral inhibition mechanisms that contribute to crowding. The connection between tES and increased cortical excitability may be mediated by the relative concentration of the inhibitory neurotransmitter GABA and the excitatory neurotransmitter glutamate within the stimulated area. Reduced GABA concentration within motor cortex following a-tDCS has been reported by multiple studies (Stagg et al., 2011; Bachtiar et al., 2018) and it is possible that tES may have a similar effect when applied to the visual cortex. Within this framework, the delayed effects of tES on contrast sensitivity (Ding et al., 2016; Nakazono et al., 2020) [and perhaps crowding (Raveendran et al., 2020)] could reflect a gradual change in GABA concentration that continues for a period after the stimulation session. However, the time course of tES effects on GABA concentration remains unclear and it is also unknown whether the effects of tES on GABA concentration are the same for the motor and visual cortices. It is worth noting that indirect evidence exists suggesting that visual cortex tES does not influence GABA (Abuleil et al., 2021). Therefore, while the effects of visual cortex tES on contrast sensitivity, crowding, and VEP amplitude are supported by our meta-analyses, the underlying mechanisms require investigation.

Meta-analyses revealed no evidence for the effectiveness of tES on visual acuity, motion perception or reaction time. The visual acuity and motion perception meta-analyses included the fewest individual experiments [two for visual acuity (Reinhart et al., 2016; Bonder et al., 2018) and three for motion perception (Zito et al., 2015; Battaglini et al., 2017; Wu et al., 2020)] with considerable variations in the visual stimuli used to measure the outcomes. The small sample combined with significant protocol differences may have limited our power to detect an effect. It is possible that measures of visual acuity and motion perception differ from contrast sensitivity and crowding in their response to tES. For motion perception, it is also possible that area MT responds to tES in a way that is distinct from that of the primary visual cortex. However, additional studies are required to fully address these questions.

The reaction time meta-analysis included seven experiments and revealed high variability across studies with two reporting longer reaction times following tES (Barbieri et al., 2016; Bonder et al., 2018), three reporting no effect (Fertonani et al., 2011; Zito et al., 2015) and two reporting shorter reaction times (Reinhart et al., 2016; van Koningsbruggen et al., 2016); one with a moderate effect size (Reinhart et al., 2016) and the other with a Hedge’s G greater than five (van Koningsbruggen et al., 2016). Reaction times can be affected by multiple variables including attention, task complexity, participant instructions, and speed accuracy trade-off. The studies included in the reaction time meta-analysis differed considerably in the types of visual stimuli and tasks employed and therefore it is perhaps not surprising that tES of cortical regions responsible for early, low-level visual processing did not produce consistent effects across studies.

Our search criteria included studies involving low and high level visual functions, however, the studies eligible for meta-analysis all focused on relatively low-level visual functions. This may be because the identified studies tended to target the primary visual cortex and therefore selected outcomes measures targeting early-stages of visual processing. However, studies not included in the meta-analysis did report NIBSs effects on face perception (Barbieri et al., 2016), visuo-motor coordination (Antal et al., 2004b,c), and attention (Laczo et al., 2012). Higher level processed such as attentive search, multisensory integration and sensory decision making are potential targets for studies exploring the potential beneficial effects of NIBS on higher level sensory function and perception.

The diverse nature of the NIBS and vision literature forced us to pool across different tES protocols, visual stimuli, and experimental designs in our meta-analyses. Therefore, our results should be interpreted with caution. In particular, a non-significant meta-analysis may reflect important variations in experimental parameters rather than no effect of the stimulation itself. The tES studies included in this review varied in terms of the stimulation devices employed and specific stimulation parameters. Unfortunately, this variation combined with the different outcome measures used across studies prevented us from conducting analyses to identify optimal visual cortex stimulation protocols. However, Table 1 does provide details of device-independent parameters such as electrode size and properties of the stimulating current to enable future analyses when a pool of more uniform studies becomes available. In addition, our inclusion of multiple independent experiments from a single publication may have amplified study-specific sources of bias. As the literature on NIBS and vision continues to develop, future meta-analyses may be able to adopt more stringent analyses criteria.

## 5. Conclusion

Our review revealed that most vision enhancement studies involving healthy populations have employed tES rather than TMS. Meta-analyses provided evidence for the effectiveness of visual cortex tES compared to sham stimulation on modulating contrast sensitivity and crowding. These effects were accompanied by evidence for a significant increase in visual cortex excitability indexed by VEP amplitude following tES. Despite the diversity of study designs in the current tES and vision literature, the results of this review indicate that tES can enhance at least some visual functions and strengthen the foundation for the application of tES in studies of vision rehabilitation. The TMS studies identified by this review also suggest that the use of TMS to enhance visual function warrants further investigation.

## Author contributions

UMB: accessing and verifying the data, conceptualization, articles’ electronic search, articles screening, meta-analysis, and draft of manuscript. JYW: accessing and verifying the data, articles screening, conceptualization, and draft of manuscript. ASYP: conceptualization, quality rating, and draft of manuscript. KWST: quality rating and draft of manuscript. BWSC: accessing and verifying the data, and data extraction. BT and AMYC: conceptualization, draft of manuscript, and project supervision. All authors had full access to the data in the study and had final responsibility for the decision to submit for publication.

## Footnotes

1
http://getdata-graph-digitizer.com/

2 Note that some studies included more than one visual functions. Hence, the sum of the total percentage exceeds 100%.

## References

[B1] Abuleil D. McCulloch D. Thompson B. (2021). Visual cortex cTBS increases mixed percept duration while a-tDCS has no effect on binocular rivalry. *PLoS One* 16:e0239349. 10.1371/journal.pone.0239349 33539443 PMC7861428

[B2] Antal A. Kincses T. Nitsche M. Bartfai O. Paulus W. (2004a). Excitability changes induced in the human primary visual cortex by transcranial direct current stimulation: direct electrophysiological evidence. *Invest. Ophthalmol. Vis. Sci.* 45 702–707. 10.1167/iovs.03-0688 14744917

[B3] Antal A. Nitsche M. Kincses T. Kruse W. Hoffmann K. Paulus W. (2004b). Facilitation of visuo-motor learning by transcranial direct current stimulation of the motor and extrastriate visual areas in humans. *Eur. J. Neurosci.* 19 2888–2892. 10.1111/j.1460-9568.2004.03367.x 15147322

[B4] Antal A. Nitsche M. Kruse W. Kincses T. Hoffmann K. Paulus W. (2004c). Direct current stimulation over V5 enhances visuomotor coordination by improving motion perception in humans. *J. Cogn. Neurosci.* 16 521–527. 10.1162/089892904323057263 15165345

[B5] Bachtiar V. Johnstone A. Berrington A. Lemke C. Johansen-Berg H. Emir U. (2018). Modulating regional motor cortical excitability with noninvasive brain stimulation results in neurochemical changes in bilateral motor cortices. *J. Neurosci.* 38 7327–7336. 10.1523/JNEUROSCI.2853-17.2018 30030397 PMC6096041

[B6] Bachtiar V. Near J. Johansen-Berg H. Stagg C. (2015). Modulation of GABA and resting state functional connectivity by transcranial direct current stimulation. *eLife* 4:e08789. 10.7554/eLife.08789 26381352 PMC4654253

[B7] Barbieri M. Negrini M. Nitsche M. Rivolta D. (2016). Anodal-tDCS over the human right occipital cortex enhances the perception and memory of both faces and objects. *Neuropsychologia* 81 238–244. 10.1016/j.neuropsychologia.2015.12.030 26744225

[B8] Battaglini L. Contemori G. Penzo S. Maniglia M. (2020a). tRNS effects on visual contrast detection. *Neurosci. Lett.* 717:134696. 10.1016/j.neulet.2019.134696 31846733

[B9] Battaglini L. Mena F. Casco C. (2020b). Improving motion detection via anodal transcranial direct current stimulation. *Restor. Neurol. Neurosci.* 38 395–405. 10.3233/RNN-201050 33016896

[B10] Battaglini L. Mena F. Ghiani A. Casco C. Melcher D. Ronconi L. (2020c). The effect of alpha tACS on the temporal resolution of visual perception. *Front. Psychol.* 11:1765. 10.3389/fpsyg.2020.01765 32849045 PMC7412991

[B11] Battaglini L. Noventa S. Casco C. (2017). Anodal and cathodal electrical stimulation over V5 improves motion perception by signal enhancement and noise reduction. *Brain Stimul.* 10 773–779. 10.1016/j.brs.2017.04.128 28487047

[B12] Battleday R. Muller T. Clayton M. Cohen Kadosh R. (2014). Mapping the mechanisms of transcranial alternating current stimulation: a pathway from network effects to cognition. *Front. Psychiatry* 5:162. 10.3389/fpsyt.2014.00162 25477826 PMC4237786

[B13] Behrens J. Kraft A. Irlbacher K. Gerhardt H. Olma M. Brandt S. (2017). Long-lasting enhancement of visual perception with repetitive noninvasive transcranial direct current stimulation. *Front. Cell Neurosci.* 11:238. 10.3389/fncel.2017.00238 28860969 PMC5559806

[B14] Bocci T. Nasini F. Caleo M. Restani L. Barloscio D. Ardolino G. (2018). Unilateral application of cathodal tDCS reduces transcallosal inhibition and improves visual acuity in amblyopic patients. *Front. Behav. Neurosci.* 12:109. 10.3389/fnbeh.2018.00109 29896093 PMC5986963

[B15] Bohotin V. Fumal A. Vandenheede M. Bohotin C. Schoenen J. (2003). Excitability of visual V1-V2 and motor cortices to single transcranial magnetic stimuli in migraine: a reappraisal using a figure-of-eight coil. *Cephalalgia* 23 264–270. 10.1046/j.1468-2982.2003.00475.x 12716343

[B16] Bonder T. Gopher D. Yeshurun Y. (2018). The joint effects of spatial cueing and transcranial direct current stimulation on visual acuity. *Front. Psychol.* 9:159. 10.3389/fpsyg.2018.00159 29515484 PMC5826080

[B17] Cabral-Calderin Y. Schmidt-Samoa C. Wilke M. (2015). Rhythmic gamma stimulation affects bistable perception. *J. Cogn. Neurosci.* 27 1298–1307. 10.1162/jocn_a_00781 25603029

[B18] Campana G. Camilleri R. Moret B. Ghin F. Pavan A. (2016). Opposite effects of high- and low-frequency transcranial random noise stimulation probed with visual motion adaptation. *Sci. Rep.* 6:38919. 10.1038/srep38919 27934947 PMC5146960

[B19] Chaieb L. Antal A. Paulus W. (2008). Gender-specific modulation of short-term neuroplasticity in the visual cortex induced by transcranial direct current stimulation. *Vis. Neurosci.* 25 77–81. 10.1017/S0952523808080097 18282312

[B20] Chen G. Zhu Z. He Q. Fang F. (2021). Offline transcranial direct current stimulation improves the ability to perceive crowded targets. *J. Vis.* 21:1. 10.1167/jov.21.2.1 33533878 PMC7862736

[B21] Clavagnier S. Thompson B. Hess R. (2013). Long lasting effects of daily theta burst rTMS sessions in the human amblyopic cortex. *Brain Stimul.* 6 860–867. 10.1016/j.brs.2013.04.002 23664756

[B22] Contemori G. Trotter Y. Cottereau B. Maniglia M. (2019). tRNS boosts perceptual learning in peripheral vision. *Neuropsychologia* 125 129–136. 10.1016/j.neuropsychologia.2019.02.001 30721741

[B23] Costa T. Costa M. Magalhaes A. Rego G. Nagy B. Boggio P. (2015a). The role of early stages of cortical visual processing in size and distance judgment: a transcranial direct current stimulation study. *Neurosci. Lett.* 588 78–82. 10.1016/j.neulet.2014.12.055 25556682

[B24] Costa T. Gualtieri M. Barboni M. Katayama R. Boggio P. Ventura D. (2015b). Contrasting effects of transcranial direct current stimulation on central and peripheral visual fields. *Exp. Brain Res.* 233 1391–1397. 10.1007/s00221-015-4213-0 25650104

[B25] Costa T. Hamer R. Nagy B. Barboni M. Gualtieri M. Boggio P. (2015c). Transcranial direct current stimulation can selectively affect different processing channels in human visual cortex. *Exp. Brain Res.* 233 1213–1223. 10.1007/s00221-015-4199-7 25600818

[B26] Costa T. Nagy B. Barboni M. Boggio P. Ventura D. (2012). Transcranial direct current stimulation modulates human color discrimination in a pathway-specific manner. *Front. Psychiatry* 3:78. 10.3389/fpsyt.2012.00078 22988446 PMC3439847

[B27] Ding Z. Li J. Spiegel D. Chen Z. Chan L. Luo G. (2016). The effect of transcranial direct current stimulation on contrast sensitivity and visual evoked potential amplitude in adults with amblyopia. *Sci. Rep.* 6:19280. 10.1038/srep19280 26763954 PMC4725886

[B28] Dong G. Wang Y. Chen X. (2020). Anodal occipital tDCS enhances spontaneous alpha activity. *Neurosci. Lett.* 721:134796. 10.1016/j.neulet.2020.134796 32006627

[B29] Downs S. Black N. (1998). The feasibility of creating a checklist for the assessment of the methodological quality both of randomised and non-randomised studies of health care interventions. *J. Epidemiol. Commun. Health* 52 377–384. 10.1136/jech.52.6.377 9764259 PMC1756728

[B30] Fertonani A. Pirulli C. Miniussi C. (2011). Random noise stimulation omproves neuroplasticity in perceptual learning. *J. Neurosci.* 31 15416–15423. 10.1523/JNEUROSCI.2002-11.2011 22031888 PMC6703532

[B31] Grabner R. Krenn J. Fink A. Arendasy M. Benedek M. (2018). Effects of alpha and gamma transcranial alternating current stimulation (tACS) on verbal creativity and intelligence test performance. *Neuropsychologia* 118 91–98. 10.1016/j.neuropsychologia.2017.10.035 29100950

[B32] Guleken Z. Eskikurt G. Karamursel S. (2020). Investigation of the effects of transcranial direct current stimulation and neurofeedback by continuous performance test. *Neurosci. Lett.* 716:134648. 10.1016/j.neulet.2019.134648 31765731

[B33] Hallett M. (2000). Transcranial magnetic stimulation and the human brain. *Nature* 406 147–150. 10.1038/35018000 10910346

[B34] Hansen B. Richard B. Andres K. Johnson A. Thompson B. Essock E. A. (2015). A cortical locus for anisotropic overlay suppression of stimuli presented at fixation. *Vis. Neurosci.* 32:E023. 10.1017/S0952523815000255 26423511

[B35] Hara T. Shanmugalingam A. McIntyre A. Burhan A. (2021). Evidence for NIBS in facilitating rehabilitation of cognitive function after stroke. *Brain Stimulation* 14:1716. 10.1016/j.brs.2021.10.420

[B36] He Q. Lin B. Zhao J. Shi Y. Yan F. Huang C. (2019). No effects of anodal transcranial direct current stimulation on contrast sensitivity function. *Restor. Neurol. Neurosci.* 37 109–118. 10.3233/RNN-180881 30856133

[B37] Heinrichs-Graham E. McDermott T. Mills M. Coolidge N. Wilson T. (2017). Transcranial direct-current stimulation modulates offline visual oscillatory activity: a magnetoencephalography study. *Cortex* 88 19–31. 10.1016/j.cortex.2016.11.016 28042984 PMC5325764

[B38] Higgins J. Thompson S. Deeks J. Altman D. (2003). Measuring inconsistency in meta-analyses. *Br. Med. J.* 327 557–560. 10.1136/bmj.327.7414.557 12958120 PMC192859

[B39] Hoy K. Bailey N. Arnold S. Windsor K. John J. Daskalakis Z. (2015). The effect of gamma-tACS on working memory performance in healthy controls. *Brain Cogn.* 101 51–56. 10.1016/j.bandc.2015.11.002 26580743

[B40] Hozo S. Djulbegovic B. Hozo I. (2005). Estimating the mean and variance from the median, range, and the size of a sample. *BMC Med. Res. Methodol.* 5:13. 10.1186/1471-2288-5-13 15840177 PMC1097734

[B41] Huang Y. Edwards M. Rounis E. Bhatia K. Rothwell J. (2005). Theta burst stimulation of the human motor cortex. *Neuron* 45 201–206. 10.1016/j.neuron.2004.12.033 15664172

[B42] Hunter M. Coffman B. Gasparovic C. Calhoun V. Trumbo M. Clark V. (2015). Baseline effects of transcranial direct current stimulation on glutamatergic neurotransmission and large-scale network connectivity. *Brain Res.* 1594 92–107. 10.1016/j.brainres.2014.09.066 25312829 PMC4358793

[B43] Kim D. Kim E. Lee C. Im C. (2019). Can anodal transcranial direct current stimulation increase steady-state visual evoked potential responses? *J. Korean Med. Sci.* 34:e285. 10.3346/jkms.2019.34.e285 31701703 PMC6838608

[B44] Kraft A. Roehmel J. Olma M. Schmidt S. Irlbacher K. Brandt S. (2010). Transcranial direct current stimulation affects visual perception measured by threshold perimetry. *Exp. Brain Res.* 207 283–290. 10.1007/s00221-010-2453-6 21046369

[B45] Laczo B. Antal A. Niebergall R. Treue S. Paulus W. (2012). Transcranial alternating stimulation in a high gamma frequency range applied over V1 improves contrast perception but does not modulate spatial attention. *Brain Stimul.* 5 484–491. 10.1016/j.brs.2011.08.008 21962982

[B46] Larcombe S. Kennard C. O’Shea J. Bridge H. (2019). No effect of anodal transcranial direct current stimulation (tDCS) over hMT plus on motion perception learning. *Front. Neurosci.* 12:1044. 10.3389/fnins.2018.01044 30705617 PMC6344419

[B47] Lau C. Tseng L. Walsh V. Hsu T. (2021). Revisiting the effects of transcranial direct current stimulation on pattern-reversal visual evoked potentials. *Neurosci. Lett.* 756:135983. 10.1016/j.neulet.2021.135983 34029648

[B48] Liew S. Santarnecchi E. Buch E. Cohen L. (2014). Non-invasive brain stimulation in neurorehabilitation: local and distant effects for motor recovery. *Front. Hum. Neurosci.* 8:378. 10.3389/fnhum.2014.00378 25018714 PMC4072967

[B49] Martin D. Moffa A. Nikolin S. Bennabi D. Brunoni A. Flannery W. (2018). Cognitive effects of transcranial direct current stimulation treatment in patients with major depressive disorder: an individual patient data meta-analysis of randomised, sham-controlled trials. *Neurosci. Biobehav. Rev.* 90 137–145. 10.1016/j.neubiorev.2018.04.008 29660416

[B50] Michael N. Gosling M. Reutemann M. Kersting A. Heindel W. Arolt V. (2003). Metabolic changes after repetitive transcranial magnetic stimulation (rTMS) of the left prefrontal cortex: a sham-controlled proton magnetic resonance spectroscopy (H-1 MRS) study of healthy brain. *Eur. J. Neurosci.* 17 2462–2468. 10.1046/j.1460-9568.2003.02683.x 12814378

[B51] Miniussi C. Harris J. Ruzzoli M. (2013). Modelling non-invasive brain stimulation in cognitive neuroscience. *Neurosci. Biobehav. Rev.* 37 1702–1712. 10.1016/j.neubiorev.2013.06.014 23827785

[B52] Moliadze V. Antal A. Paulus W. (2010). Boosting brain excitability by transcranial high frequency stimulation in the ripple range. *J. Physiol.* 588 4891–4904. 10.1113/jphysiol.2010.196998 20962008 PMC3036186

[B53] Moreno L. Senra H. Lewis P. Moreno N. Linhares J. Santana R. (2020). Cost-effectiveness of basic vision rehabilitation (The basic VRS-effect study): study protocol for a randomised controlled trial. *Ophthalmic Physiol. Opt.* 40 350–364. 10.1111/opo.12665 31989690

[B54] Nakazono H. Ogata K. Takeda A. Yamada E. Kimura T. Tobimatsu S. (2020). Transcranial alternating current stimulation of α but not β frequency sharpens multiple visual functions. *Brain Stimul.* 13 343–352. 10.1016/j.brs.2019.10.022 31711878

[B55] Nitsche M. Paulus W. (2000). Excitability changes induced in the human motor cortex by weak transcranial direct current stimulation. *J. Physiol.* 527 633–639. 10.1111/j.1469-7793.2000.t01-1-00633.x 10990547 PMC2270099

[B56] Nitsche M. Paulus W. (2001). Sustained excitability elevations induced by transcranial DC motor cortex stimulation in humans. *Neurology* 57 1899–1901. 10.1212/WNL.57.10.1899 11723286

[B57] Nitsche M. Nitsche M. Klein C. Tergau F. Rothwell J. Paulus W. (2003). Level of action of cathodal DC polarisation induced inhibition of the human motor cortex. *Clin. Neurophysiol.* 114 600–604. 10.1016/S1388-2457(02)00412-1 12686268

[B58] Olma M. Dargie R. Behrens J. Kraft A. Irlbacher K. Fahle M. (2013). Long-term effects of serial anodal tDCS on motion perception in subjects with occipital stroke measured in the unaffected visual hemifield. *Front. Hum. Neurosci.* 7:314. 10.3389/fnhum.2013.00314 23805097 PMC3690540

[B59] Page M. McKenzie J. Bossuyt P. Boutron I. Hoffmann T. Mulrow C. (2021). The PRISMA 2020 statement: an updated guideline for reporting systematic reviews. *Int. J. Surg.* 88:105906. 10.1016/j.ijsu.2021.105906 33789826

[B60] Pascual-Leone A. Tormos J. Keenan J. Tarazona F. Canete C. Catala M. (1998). Study and modulation of human cortical excitability with transcranial magnetic stimulation. *J. Clin. Neurophysiol.* 15 333–343. 10.1097/00004691-199807000-00005 9736467

[B61] Pascualleone A. Vallssole J. Wassermann E. Hallett M. (1994). Responses to rapid-rate transcranial magnetic stimulation of the human motor cortex. *Brain* 117 847–858. 10.1093/brain/117.4.847 7922470

[B62] Peters M. Thompson B. Merabet L. Wu A. Shams L. (2013). Anodal tDCS to V1 blocks visual perceptual learning consolidation. *Neuropsychologia* 51 1234–1239. 10.1016/j.neuropsychologia.2013.03.013 23562964

[B63] Pirulli C. Fertonani A. Miniussi C. (2013). The role of timing in the induction of neuromodulation in perceptual learning by transcranial electric stimulation. *Brain Stimul.* 6 683–689. 10.1016/j.brs.2012.12.005 23369505

[B64] Plow E. Obretenova S. Fregni F. Pascual-Leone A. Merabet L. (2012). Comparison of visual field training for hemianopia with active versus sham transcranial direct cortical stimulation. *Neurorehabil. Neural Repair.* 26 616–626. 10.1177/1545968311431963 22291042

[B65] Ranieri F. Coppola G. Musumeci G. Capone F. Di Pino G. Parisi V. (2019). Evidence for associative plasticity in the human visual cortex. *Brain Stimul.* 12 705–713. 10.1016/j.brs.2019.01.021 30773491

[B66] Raveendran R. Tsang K. Tiwana D. Chow A. Thompson B. (2020). Anodal transcranial direct current stimulation reduces collinear lateral inhibition in normal peripheral vision. *PLoS One* 15:e0232276. 10.1371/journal.pone.0232276 32374787 PMC7202594

[B67] Reed T. Kadosh R. (2018). Transcranial electrical stimulation (tES) mechanisms and its effects on cortical excitability and connectivity. *J. Inherit. Metab. Dis.* 41 1123–1130. 10.1007/s10545-018-0181-4 30006770 PMC6326965

[B68] Reinhart R. Xiao W. McClenahan L. Woodman G. (2016). Electrical stimulation of visual cortex can immediately improve spatial vision. *Curr. Biol.* 26 1867–1872. 10.1016/j.cub.2016.05.019 27374337 PMC4961578

[B69] Richard B. Johnson A. Thompson B. Hansen B. (2015). The effects of tDCS across the spatial frequencies and orientations that comprise the contrast sensitivity function. *Front. Psychol.* 6:1784. 10.3389/fpsyg.2015.01784 26640448 PMC4661264

[B70] Schaeffner L. Welchman A. (2019). The mixed-polarity benefit of stereopsis arises in early visual cortex. *J. Vis.* 19:9. 10.1167/19.2.9 30779843 PMC6380879

[B71] Sczesny-Kaiser M. Beckhaus K. Dinse H. Schwenkreis P. Tegenthoff M. Hoffken O. (2016). Repetitive transcranial direct current stimulation induced excitability changes of primary visual cortex and visual learning effects- a pilot study. *Front. Behav. Neurosci.* 10:116. 10.3389/fnbeh.2016.00116 27375452 PMC4891342

[B72] Somer E. Allen J. Brooks J. Buttrill V. Javadi A. (2020). Theta phase-dependent modulation of perception by concurrent transcranial alternating current stimulation and periodic visual stimulation. *J. Cogn. Neurosci.* 32 1142–1152. 10.1162/jocn_a_01539 32013685

[B73] Spiegel D. Hansen B. Byblow W. Thompson B. (2012). Anodal transcranial direct current stimulation reduces psychophysically measured surround suppression in the human visual cortex. *PLoS One* 7:e36220. 10.1371/journal.pone.0036220 22563485 PMC3341359

[B74] Spiegel D. Li J. Hess R. Byblow W. Deng D. Yu M. (2013). Transcranial direct current stimulation enhances recovery of stereopsis in adults with amblyopia. *Neurotherapeutics* 10 831–839. 10.1007/s13311-013-0200-y 23857313 PMC3805870

[B75] Stagg C. Bachtiar V. Johansen-Berg H. (2011). The role of GABA in human motor learning. *Curr. Biol.* 21 480–484. 10.1016/j.cub.2011.01.069 21376596 PMC3063350

[B76] Stagg C. Best J. Stephenson M. O’Shea J. Wylezinska M. Kincses Z. (2009). Polarity-sensitive modulation of cortical neurotransmitters by transcranial stimulation. *J. Neurosci.* 29 5202–5206. 10.1523/JNEUROSCI.4432-08.2009 19386916 PMC6665468

[B77] Terney D. Chaieb L. Moliadze V. Antal A. Paulus W. (2008). Increasing human brain excitability by transcranial high-frequency random noise stimulation. *J. Neurosci.* 28 14147–14155. 10.1523/JNEUROSCI.4248-08.2008 19109497 PMC6671476

[B78] Thompson B. Aaen-Stockdale C. Koski L. Hess R. F. (2009). A double dissociation between striate and extrastriate visual cortex for pattern motion perception revealed using rTMS. *Hum. Brain Mapp.* 30 3115–3126. 10.1002/hbm.20736 19224619 PMC6870809

[B79] Thompson B. Deblieck C. Wu A. Iacoboni M. Liu Z. (2016). Psychophysical and rTMS evidence for the presence of motion opponency in human V5. *Brain Stimul.* 9 876–881. 10.1016/j.brs.2016.05.012 27342938 PMC5143189

[B80] Thompson B. Mansouri B. Koski L. Hess R. (2008). Brain plasticity in the adult: modulation of function in amblyopia with rTMS. *Curr. Biol.* 18 1067–1071. 10.1016/j.cub.2008.06.052 18635353

[B81] Valentine J. Pigott T. Rothstein H. (2010). How many studies do you need?: a primer on statistical power for meta-analysis. *J. Educ. Behav. Stat.* 35 215–247. 10.3102/1076998609346961

[B82] van Koningsbruggen M. Ficarella S. Battelli L. Hickey C. (2016). Transcranial random-noise stimulation of visual cortex potentiates value-driven attentional capture. *Soc. Cogn. Affect. Neurosci.* 11 1481–1488. 10.1093/scan/nsw056 27107297 PMC5015805

[B83] Van Meel C. Daniels N. Op de Beeck H. Baeck A. (2016). Effect of tDCS on task relevant and irrelevant perceptual learning of complex objects. *J. Vis.* 16:13. 10.1167/16.6.13 27096945

[B84] Waterston M. Pack C. (2010). Improved discrimination of visual stimuli following repetitive transcranial magnetic stimulation. *PLoS One* 5:e10354. 10.1371/journal.pone.0010354 20442776 PMC2860988

[B85] Wu D. Li C. Liu N. Xu P. Xiao W. (2020). Visual motion perception improvements following direct current stimulation over V5 are dependent on initial performance. *Exp. Brain Res.* 238 2409–2416. 10.1007/s00221-020-05842-7 32776173

[B86] Wunder S. Hunold A. Fiedler P. Schlegelmilch F. Schellhorn K. Haueisen J. (2018). Novel bifunctional cap for simultaneous electroencephalography and transcranial electrical stimulation. *Sci. Rep.* 8:7259. 10.1038/s41598-018-25562-x 29740054 PMC5940899

[B87] Zito G. Senti T. Cazzoli D. Muri R. Mosimann U. Nyffeler T. (2015). Cathodal HD-tDCS on the right V5 improves motion perception in humans. *Front. Behav. Neurosci.* 9:257. 10.3389/fnbeh.2015.00257 26441582 PMC4585077

