# Supplementary material for: Can visual cortex non-invasive brain stimulation improve normal visual function? A systematic review and meta-analysis
Source: Front Neurosci. 2023 Mar 2;17:1119200. doi: 10.3389/fnins.2023.1119200 (PMC10017867; doi:10.3389/fnins.2023.1119200)
Supplement: Supplementary file 1 [file Data_Sheet_1.docx]

**Appendix 1: Search strategy**

| **Search Terms** | **Search Themes** | |
| --- | --- | --- |
|  | ***Brain area*** | ***Noninvasive brain stimulation (NIBS)*** |
|  | “Primary visual cortex”  “V1”  “Extrastriate cortex”  “V2”  “V3”  “V3a”  “V4”  “V5”  ”Middle temporal”  “MT”  “Medial superior temporal”  “MST”  “Occipital pole”  “Occipital lobe” | “Transcranial direct current stimulation”  “tDCS”  “Transcranial alternating current stimulation”  “tACS”  “Transcranial random noise stimulation”  “tRNS”  “Transcranial magnetic stimulation”  “TMS”  “Repetitive transcranial magnetic stimulation”  “rTMS”  “Continuous theta burst stimulation”  “cTBS”  “Theta burst stimulation”  “TBS” |

**Appendix 2: Reasons for the studies excluded from the meta-analysis**

| **S/N** | **Study** | **Visual functions** | **Reason(s)** |
| --- | --- | --- | --- |
| 1 | Abuleil 2021 (59) | Binocular rivalry, mixed percept duration | The only paper in category for visual function (for both tDCS stimulation and **TMS** **stimulation (cTBS))** |
| 2 | Antal 2004 (60) | Visuo-motor coordination | No sham data |
| 3 | Antal 2004 (63) | VEP | No sham group |
| 4 | Antal 2004 (61) | Visuo-motor coordination | The only paper in category (visual function) |
| 5 | Battaglini 2020 (64) | Visual temporal resolution | The only paper in category (visual function) |
| 6 | Battaglini 2020 (65) | Motion perception | No appropriate data for meta-analysis (only a sensitivity parameter - d’prime was provided and no raw data were provided by the corresponding author) |
| 7 | Bocci 2018 (66) | VA and VEP | No sham group |
| 8 | Cabral-Calderin 2015 (67) | Bistable perception | The only paper in category (visual function) |
| 9 | Campana 2016 (68) | Visual motion adaptation | The only paper in category (visual function) |
| 10 | Chaieb 2008 (69) | VEP, CS | No appropriate data for meta-analysis (only percentage change were reported, and no raw data were provided by the corresponding author) |
| 11 | Costa 2015 (70) | Size and distance of judgement | The only paper in category (visual function) |
| 12 | Costa 2015 (71) | Visual field | The only paper in category (visual function) |
| 13 | Costa 2015 (72) | VEP | Sweep VEP instead of pattern/ flash VEP was used. Parameters reported were very different from other studies (amplitude of suprathreshold) |
| 14 | Costa 2012 (73) | Color discrimination | The only paper in category (visual function) |
| 15 | Hanson 2015 (74) | Overlay and surround suppression | No sham group |
| 16 | Heinrichs-Graham 2017 (75) | Oscillatory activity | The only paper in category (visual function) |
| 17 | Kim 2019 (76) | VEP (steady-state) | No appropriate data for meta-analysis (signal-to-noise ratio and no raw data were provided by the corresponding author) |
| 18 | Laczo 2012 (62) | CS, visual attention | No appropriate data for meta-analysis (only percentage change were reported and no raw data were provided by the corresponding author) |
| 19 | Larcombe 2019 (77) | Motion perception | Combined tDCS with visual training was reported (no data for pure tDCS effect) |
| 20 | Peters 2013 (78) | CS, VEP | No data for the acute effect (i.e. immediately after stimulation) |
| 21 | Pirulli 2013 (79) | Orientation and discrimination | The only paper in category (visual function) |
| 22 | Ranieri 2019 (80) | VEP | The only paper in category (**TMS stimulation**) |
| 23 | Richard 2015 (81) | CS | No sham group |
| 24 | Schaeffner 2019 (82) | Contrast polarity and depth perception | The only paper in category (visual function and **TMS stimulation**) |
| 25 | Sczesny-Kaiser 2016 (83) | VEP | VEP parameters reported were very different from other papers (amp ratio (A2s/A1)) |
| 26 | Somer 2020 (84) | RT | No appropriate data for meta-analysis (no pre-post data, no sham data and no raw data were provided by the corresponding author) |
| 27 | Spiegel 2012 (85) | Surround suppression | The only paper in category (visual function) |
| 28 | Thompson 2008 (26) | CS | The only paper in category (**rTMS stimulation**) |
| 29 | Van Meel 2016 (86) | Object recognition, orientation judgement | The only paper in category (visual function) |
| 30 | Waterston 2010 (87) | CS (course orientation and discrimination) at peripheral vision | The only paper in category (visual function and **rTMS stimulation**) |
| 31 | Wunder 2018 (88) | VEP | No sham group |

***Abbreviations*:** CS (contrast sensitivity); RT (reaction time); VA (visual acuity); VEP (visual evoked potentials)

Bolded text refers to the study involving transcranial magnetic stimulation (TMS), including rTMS (repetitive transcranial magnetic stimulation) and cTBS (continuous theta burst stimulation).
